# Nanogels and Microgels: Fundamental Studies, Applications, and Emerging Trends

**DOI:** 10.3390/gels12100890

**Published:** 2026-10-01

**Authors:** Jiaxing Yuan, Guang Chen

**Affiliations:** 1 Advanced Materials Thrust, Function Hub, The Hong Kong University of Science and Technology (Guangzhou), Guangzhou 511453, China; 2 Department of Advanced Manufacturing and Robotics, College of Engineering, Peking University, Beijing 100871, China

## 1. Introduction

Polymer gels constitute a versatile class of three-dimensional, cross-linked macromolecular networks that have fostered interdisciplinary interest owing to their tunable physicochemical properties and structural versatility [1]. Their distinctive network topology allows them to function as stimuli-responsive platforms capable of undergoing reversible conformational changes in response to environmental stimuli. This intrinsic adaptability underpins their utility across a spectrum of emerging technologies. In recent years, gels have increasingly been used in various domains, including targeted drug delivery, tissue-engineered scaffolds, and flexible bioelectronic interfaces [2].

While conventional gels operate at the macroscopic scale, spatial confinement of these networks gives rise to microgels and nanogels, which are primarily distinguished by their characteristic length scales. Microgels, with dimensions ranging from 1 to 1000 μm, occupy an intermediate zone between macroscopic gels and isolated polymer chains. They offer highly customized porosity and localized environmental sensitivity, making them promising candidates for functional surface coatings, adaptive sensing platforms, and precision-targeted therapeutic delivery vehicles. In contrast, nanogels, confined to the sub-micrometer regime (1–100 nm), are distinguished by their exceptionally high surface-area-to-volume ratios [3,4]. Owing to their diminutive dimensions, nanogels exhibit markedly faster responses relative to their bulk hydrogel counterparts [5,6]. Moreover, their nano- and microscale dimensions enable them to efficiently navigate biological barriers while evading rapid clearance by the reticuloendothelial system. This synergy of swift responsiveness and high biopermeability makes microgels and nanogels highly promising for systemic drug delivery and next-generation platforms that require real-time, adaptive structural modulation [2,7].

In recognition of these emerging developments, *Gels* has launched the Special Issue “Nanogels and Microgels: Fundamental Studies, Applications, and Emerging Trends.” This collection consolidates recent advances and fosters scholarly dialogue across the diverse community, investigating the structure, dynamics, and functions of nanogels and microgels. The Special Issue comprises eight insightful contributions—including three research articles and five review papers—that showcase the breadth and ingenuity driving gel research. These contributions span a broad landscape, encompassing fundamental interfacial science, biomedical engineering, and smart materials, thereby underscoring the multidisciplinary character of the field. In the following section, we provide an overview of each study, delineating their research objectives, key findings, and implications for the future study of gels.

## 2. Overview of the Papers Published in This Special Issue

“Effect of Polymer Network Architecture on Adsorption Kinetics at Liquid–Liquid Interfaces: A Comparison Between Poly(NIPAM-co-AA) Copolymer Microgels and Interpenetrating Network Microgels” by Komarova et al. investigates a fundamental application of polymer gels in interfacial science and emulsion stabilization. The authors examine the unique internal architecture and adsorption behavior of poly(NIPAM-co-AA) microgels, comparing random copolymer structures against interpenetrating polymer networks (IPNs), which form distinctive topological arrangements that endow them with highly tunable deformability and stimuli-responsiveness. While these soft microgels effectively migrate to liquid–liquid boundaries to stabilize smart emulsions, their different internal entanglements create distinct variations in their spreading kinetics and structural reorganization. The researchers systematically track the multi-step adsorption process—from bulk diffusion to interfacial deformation—at the oil–water interface. Their work demonstrates that network topology profoundly dictates spreading speed, with specific architectures achieving interfacial saturation and structural equilibrium significantly faster. The study’s findings are relevant for the pharmaceutical, cosmetic, and food industries, showing how gel architecture can be precisely leveraged to control emulsion stability.

“In Situ Gelling of TPZ-Loaded Nanogel for Sustained Intratumoral Delivery and Enhanced Cancer Immunotherapy” by Li et al. details the development of a thermosensitive nanogel system to address the limitations of the hypoxia-activated anticancer agent tirapazamine (TPZ) in cancer therapy. While TPZ offers selective cytotoxicity in hypoxic tumor microenvironments and can be delivered via intratumoral injection, its clinical efficacy is hampered by rapid diffusion and clearance from the injection site, leading to poor drug retention and suboptimal therapeutic outcomes. To overcome these issues, the authors designed a formulation that leverages a sol–gel phase transition to form an in situ depot upon injection. Mechanistically, the treatment not only inhibited tumor cell proliferation and promoted apoptosis and anti-angiogenesis but also markedly enhanced antitumor immune responses, ultimately inducing extensive ischemic necrosis. These findings highlight this nanogel platform’s therapeutic potential for cancer therapy.

“Protocol-Dependent Effects on Colloidal Characterization and Drug Loading/Release Analysis of Thermosensitive PNIPAM-co-COOH Microgels” by López-Molina et al. investigates the critical impact of analytical protocols on the characterization and drug delivery performance of thermosensitive PNIPAM-co-COOH microgels, establishing a robust framework with which to minimize experimental bias. While these smart microgels are widely used as nanocarriers because of their tunable collapse and swelling behavior, the authors identify that standard characterization methods introduce significant artifacts, leading to underestimation of the collapsed hydrodynamic radius by 18% at 43 °C owing to thermal convection. To address this issue, they systematically compared multiple orthogonal techniques, demonstrating that drift-corrected 3D-DLS combined with SLS can provide a consistent and accurate description of temperature-induced collapse, pH-dependent swelling, and core–corona architecture. The study’s findings are significant for pharmaceutics and nanomedicine, showing how targeted methodological refinements can reduce experimental artifacts and provide a reliable characterization framework that may be extended to other soft nanocarriers.

The review paper “Clinical Applications and Therapeutic Potential of Nano-Bio Fusion Gel in Oral Soft Tissue Therapy: A Critical Narrative Review” by Kammerhofer et al. focuses on the emerging clinical applications of bioadhesive polymer gels in periodontology and oral medicine. The authors examine the therapeutic potential and delivery characteristics of Nano-Bio Fusion (NBF) gingival gel—a nano-formulated bioadhesive matrix incorporating propolis, vitamin C, and vitamin E—which forms a mucosal-adherent network that endows it with prolonged local retention and potent tissue-modulating properties. While conventional topical therapies effectively manage oral inflammation, their therapeutic efficacy is frequently compromised by rapid salivary washout, mechanical clearance, and adverse long-term effects. To address these issues, the authors conducted a comprehensive review to evaluate this nano-formulated alternative across a spectrum of oral pathologies, including chronic periodontitis, gingivitis, and postsurgical alveolar osteitis. Clinical evaluations demonstrated that this mucoadhesive formulation significantly improves periodontal attachment levels and reduces postoperative pain, leading to therapeutic outcomes comparable to those of standard antimicrobial regimens. These findings show how nano-formulated bioadhesive gel chemistry can be leveraged to overcome oral delivery barriers and promote targeted soft-tissue regeneration in a safe, biocompatible manner.

“Polymeric Nanogels for Skin Applications”, a review by Silva et al., comprehensively covers recent developments relating to nanohydrogels. By shrinking moisture-rich, biocompatible hydrogel networks down to the nanoscale (10–200 nm), researchers have engineered smart, sponge-like carriers capable of slipping deep into the epidermal layers. What makes these nanogels truly revolutionary is their “stimuli-responsive” design; they can be custom-tailored to release therapeutics precisely when triggered by the subtle heat, acidity, or oxidative stress unique to inflamed tissues. Beyond simply delivering drugs, these advanced systems—particularly those utilizing natural biopolymers or integrated nanozymes—actively participate in the healing process by neutralizing harmful free radicals and restoring local immune balance. This review clearly maps out how these highly tunable, intelligent nanonetworks are paving the way toward a new era of adaptive, personalized transdermal medicine.

Tong et al.’s review, “Emerging Gel Technologies for Atherosclerosis Research and Intervention”, explores the transformative potential of gel-based technologies in revolutionizing both the fundamental investigation and targeted treatment of atherosclerosis. While conventional systemic drugs and mechanical interventions (such as stenting) remain constrained by off-target side effects, inadequate local delivery, and late-stage restenosis, highly customizable hydrogels and nanogels serve as a multifaceted alternative. The authors systematically review the utility of these three-dimensional polymer networks across multiple scales: from engineered in vitro disease models that faithfully recapitulate plaque microenvironments to macroscopic therapeutic depots optimized for localized, sustained drug release. Furthermore, the review highlights the emergence of stimuli-responsive nanogels and inorganic-organic theranostic composites designed to navigate systemic circulation and selectively activate upon encountering the acidic, oxidatively stressed, or enzyme-rich milieu characteristic of atherosclerotic lesions. Ultimately, the study underscores how intelligently engineered polymer gel platforms—soon to be bolstered by AI-guided design—are uniquely positioned to deliver unprecedented spatial and temporal precision in next-generation cardiovascular care.

“Polymer Network-Based Nanogels and Microgels: Design, Classification, Synthesis, and Applications in Drug Delivery”, a review by Sutradhar et al., explores the design, synthesis, and multifaceted biomedical applications of polymer-network-based nanogels and microgels as next-generation drug delivery platforms. The authors classify these highly tunable, water-swollen networks according to their crosslinking mechanisms, material origins, and sophisticated stimulus-responsive behaviors. By comparing advanced synthesis techniques—such as inverse microemulsion and radiation-induced polymerization—the study illustrates how these systems are engineered to safely encapsulate diverse therapeutics (from small molecules to proteins and nucleic acids) and successfully penetrate formidable physiological obstacles like the blood–brain barrier. Furthermore, the review highlights the potential of these smart soft materials across a wide array of clinical applications. The authors emphasize that emerging solutions like microfluidic synthesis and AI-guided personalized design will solidify nanogels and microgels as indispensable tools in the future of precision nanomedicine.

Kim’s review, “Hybrid Systems of Gels and Nanoparticles for Cancer Therapy: Advances in Multifunctional Therapeutic Platforms”, summarizes the recent advancements in and therapeutic potential of gel–nanoparticle hybrid systems in cancer treatment. While various nanoparticles (such as silica, quantum dots, and metal–organic frameworks) can passively accumulate in tumors via the enhanced permeability and the retention effect, their clinical efficacy is often severely limited by their poor biocompatibility, rapid clearance by the reticuloendothelial system, and inadequate drug-loading capacity. To overcome these barriers, researchers are integrating nanoparticles into three-dimensional gel networks. The resulting hybrid platforms synergize the strengths of both components: the gel matrix dramatically enhances biocompatibility, prolongs localized tumor retention through tissue adhesion, and enables stimulus-responsive (e.g., to pH, temperature, and near-infrared light) controlled drug release, while the embedded nanoparticles confer distinct properties for targeted chemotherapy, photothermal therapy (PTT), and immunomodulation. Despite promising outcomes, the author remarks that realizing the full commercial potential of these multimodal platforms will still require advanced, scalable synthesis techniques; rigorous long-term in vivo safety profiling; and standardized regulatory frameworks.

## 3. Conclusions and Outlook

The eight works presented in this Special Issue collectively show that polymeric gels—encompassing nanogels, microgels, and hybrid networks—have matured into indispensable, highly adaptable platforms for interfacial science and advanced therapeutic delivery. Whether governing adsorption kinetics at liquid–liquid interfaces or functioning as biomimetic scaffolds, the distinctive three-dimensional architectures of these soft materials afford exceptional structural versatility. Through the rational design of networks that respond dynamically to pathological microenvironments or external stimuli, researchers have achieved unprecedented spatiotemporal control over drug release. Moreover, whether applied as gels for periodontal regeneration, transdermal nanohydrogels for chronic skin inflammation, or nanoparticle-integrated hybrid systems for cancer therapy, these intelligent materials consistently overcome formidable physiological barriers, highlighting their broad translational potential.

Despite these remarkable successes, translating smart gel materials from the laboratory bench to widespread clinical application remains challenging. To bridge this gap, researchers must prioritize transitioning from small-scale batch synthesis to continuous, scalable production. The widespread adoption of microfluidic technologies and 3D bioprinting will be critical to ensuring precise control over particle size, cross-linking density, and batch-to-batch consistency, as required for clinical-grade manufacturing. Furthermore, we foresee that the next generation of gel technologies will be augmented by artificial intelligence. AI-driven research will dramatically accelerate the rational design and screening of novel gel structures. We believe that as multidisciplinary collaborations continue to deepen, polymeric gel technologies are poised to enter a new era defined by adaptive, high-precision, and personalized medicine. In conclusion, this Special Issue reveals microgel and nanogel research as a dynamic, interdisciplinary field with profound societal relevance, particularly in biomedical science. By bridging fundamental polymer science with application-oriented development, the contributions assembled here push the frontiers of gel materials in novel and impactful directions. We anticipate that the insights presented will inspire continued innovation and help catalyze next-generation technologies with far-reaching global implications.
